# Boosting Texture-Based Classification by Describing Statistical Information of Gray-Levels Differences

**DOI:** 10.3390/s19051048

**Published:** 2019-03-01

**Authors:** Óscar García-Olalla, Laura Fernández-Robles, Enrique Alegre, Manuel Castejón-Limas, Eduardo Fidalgo

**Affiliations:** 1 Department of Electrical, Systems and Automation, Universidad de León, 24007 León, Spain; ogaro@unileon.es (O.G.-O.); ealeg@unileon.es (E.A.); 2 Department of Mechanical, Computer Science and Aerospace Engineering, Universidad de León, 24007 León, Spain; l.fernandez@unileon.es (L.F.-R.); mcasl@unileon.es (M.C.-L.); 3 Spanish National Cybersecurity Institute (INCIBE), 24005 León, Spain

**Keywords:** CLOSIB, statistical information of gray-levels differences, Local Binary Patterns, texture classification, texture description, Visual Sensors

## Abstract

This paper presents a new texture descriptor booster, Complete Local Oriented Statistical Information Booster (CLOSIB), based on statistical information of the image. Our proposal uses the statistical information of the texture provided by the image gray-levels differences to increase the discriminative capability of Local Binary Patterns (LBP)-based and other texture descriptors. We demonstrated that Half-CLOSIB and M-CLOSIB versions are more efficient and precise than the general one. H-CLOSIB may eliminate redundant statistical information and the multi-scale version, M-CLOSIB, is more robust. We evaluated our method using four datasets: KTH TIPS (2-a) for material recognition, UIUC and USPTex for general texture recognition and JAFFE for face recognition. The results show that when we combine CLOSIB with well-known LBP-based descriptors, the hit rate increases in all the cases, introducing in this way the idea that CLOSIB can be used to enhance the description of texture in a significant number of situations. Additionally, a comparison with recent algorithms demonstrates that a combination of LBP methods with CLOSIB variants obtains comparable results to those of the state-of-the-art.

## 1. Introduction

Texture description is one of the main and active fields of research in computer vision [1] and it has a high impact in several research areas connected to image processing and pattern recognition. Texture description is a challenging task that deals with several open problems, e.g., highly discriminate inter-class textures while achieving robustness to intra-class variations. Moreover, the same texture can be displayed in different images under very different appearance due to modifications in the luminance, quality of the camera, snapshot angle, occlusions, and so on. It is for all of these reasons that texture description is still an open problem. Many experimental datasets [2,3] have been created to analyze and fairly compare new methods about texture description. Two of such datasets are UIUC and USPTex, and recent proposals on computer vision are tested on them. Among the most relevant ones, in 2017, Backes et al. [4] obtained discriminative texture signatures by using the LBP approach and fractal dimension to calculate features from the LBP sources of information resulting in an accuracy of 72.50% and 86.52% for the UIUC and USPTex respectively. Florindo et al. [5] in 2016 computed a connectivity index within a local image neighborhood that corresponded to the number of pixels more closely related to the central pixel yielding a success rate of 88.6% on UIUC dataset. In 2017, Cernadas et al. [6] tested different normalization algorithms for color texture classification on USPTex dataset achieving an accuracy of 95.6%. Casanova et al. [7] in 2016 expressed the complexity of the relations among color channels and obtained a hit rate of 97.04% on USPTex dataset. A wide range of applications needs an appropriate texture description of the regions of interest, such as quality control in factories [8], pedestrian detection in crowded streets [9], medical image diagnoses [10] or geographical analyses of optical remote sensing (RS) images [11].

Material recognition is an important field of visual recognition. Even though it differs from texture recognition since one pattern can be made of different materials, texture features are commonly used for material description. Plenty of industries need quality control of their manufactured products, and the use of cameras multiply the speed of this process, avoiding the possibility of subjective interpretations by an operator. In this line of work, González et al. [12] proposed an adaptative method based on pattern spectrum texture descriptor, in which the structural element shape depends on a distance criterion using euclidean and geodesic metrics. Alegre et al. [13] used texture features based on the Laws filters information to evaluate the surface roughness of inserts in milling head tools. In this paper, we evaluate our proposed method for material recognition purposes using KTH Tips2-a [14] dataset. This dataset, created by Caputo et al. and presented in [15], is very popular for material recognition. Chen et al. [16] proposed a method called Weber Local Descriptor (WLD) based on the Weber’s Law that achieved a 64.7% of hit rate on this dataset. Hussain et al. in 2012 presented a method called Local Quantified Patterns (LQP) [17] which yielded an accuracy of 64.2% on the same dataset whereas Hafiane et al. [18] in 2015 achieved a 70.3% of accuracy using a method, Adaptive Median Binary Pattern (AMBP), based on LBP. Due to the high intra-class variation of the classes in KTH TIPS2-a, the accuracy obtained in different works of the literature for this dataset is still moderate. Face recognition is another interesting field for many commercial applications in which texture description has demonstrated to be very useful [19]. Faces are highly variable even though the geometry and appearance are not too complicated. Due to the difficulty of the face recognition task, the number of techniques proposed is large and diverse [20,21]. In this paper, we used the JApanese Female Facial Expression (JAFFE) dataset [22] which was developed by Lyons et al. in 1998 and is still extensively used not only for facial expression but also for facial recognition tasks [23]. Rangaswamy et al. proposed a new technique for face recognition based on a fusion of Wavelet and Fourier features [24]. In the same line of work, Wan et al. [25] achieved a 79% of hit rate using a new method called Quasi-Singular Value Decomposition Random Weight Network (Q-SVD + RWN). Zang et al. [26] yielded an accuracy equal to 86.42%, employing Elastic Preserving Projections (EPP) algorithm.

In recent years, local descriptors have been widely used for multiple problems with very promising results. LBP is one of the most popular methods since Ojala et al. introduced it in Ref. [27]. It presents a low computational cost and complexity and a high capability to describe the texture. Nowadays, there are plenty of research groups studying and proposing new methods based on LBP. The original research group at Oulu University proposed several modifications such as Local Binary Patterns Histogram Fourier Features [28], the spatio-temporal LBP –Volume LBP (VLBP) and LBP on Three Orthogonal Planes (LBP-TOP)—[29] or Lineal Configuration Pattern model (LCP) [30]. Guo and his research group at Honk Kong University developed several variants aiming to add extra information to LBP descriptors. Some of their methods can be found in [31,32,33] and are briefly explained in the related works section. Specifically, Completed LBP (CLBP) has proven to be one of the best performing non-parametric texture description operators by independent authors [34]. Many more variants of LBP exist; we refer the reader to a recent review on this topic for further details [35]. However, none of the previous works deals with the study of the variations of the gray-level differences at several orientations of the image. García-Olalla et al. studied methods that make use of the statistical information of the image and combine them with LBP [36,37,38], developing a new booster algorithm which improved previous results [39]. Although this booster outperformed LBP and other state-of-the-art methods, it is very specific and is only able to evaluate one statistical order, the mean of the gray level differences along several orientations, for a unique neighborhood configuration.

In this paper, we present a novel method following the work carried out in Ref. [39] that we name CLOSIB, that stands for Complete Local Oriented Statistical Information Booster [40]. CLOSIB is a new texture booster which extracts statistical information of the gray-scale differences in several orientations of the image. Therefore, it can be fused with other descriptors in order to comprise statistical information of the image. We compare our method versus LBP and three LBP-based methods (Adaptive LBP (ALBP) [31], LBP Variance (LBPV) [32] and Complete LBP (CLBP) [33]) due to the high confidence and performance of these methods in a wide application range. Furthermore, we propose and discuss three new variants of CLOSIB based on multi-scale and feature selection: Half CLOSIB (H-CLOSIB), Multi-scale CLOSIB (M-CLOSIB) and Half Multi-scale CLOSIB (HM-CLOSIB). In order to evaluate the performance of CLOSIB and its efficiency in combination with LBP-based descriptors, we tested our method with four texture datasets. Specifically, KTH Tips2-a [15] for material recognition, UIUC [41] and USPTex [42] for general texture classification, and JAFFE [22] for face recognition. At the moment of the submission of this paper, we have already published results using CLOSIB combined with HOG features [43], where we worked on textile retrieval from images obtained on indoor environments. Regardless of this publication [43], where we applied CLOSIB to the mentioned specific problem, in this paper, we present and explain, for the first time, the whole method and context. We evaluate it on four publicly available datasets, comparing it against 18 handcrafted and three deep learning-based state-of-the-art approaches.

## 2. Related Works

In this section we review the four LBP variants studied. Due to the number of parameters introduced in our study, we include a table of notations, Table 1, to improve the equation readability.

### 2.1. Descriptors Based on LBP

#### 2.1.1. Local Binary Patterns (LBP)

LBP [44] describes the texture of gray-scale images extracting their local spatial structure and using a very simple computation. For each pixel, a pattern code is obtained by comparing its value with the value of its neighbors:

(1) $$LBP_{P,R}=\sum_{p=0}^{P-1}s\left(g_{p}-g_{c}\right)2^{p}\;,\;s\left(x\right)=\left\{\begin{array}{cc} 1 & \mathrm{if}x\geq 0\\ 0 & \mathrm{if}x<0 \end{array}\right.$$

where $g_{c}$ is the gray value of the central pixel, $g_{p}$ is the value of its neighbor *p*, *P* is the number of neighbors and *R* is the radius of the neighborhood. An image is described by means of a histogram of the LBP values at each pixel of the image. Ojala et al. [44] introduced the rotation invariant uniform operator, $LBP_{P,R}^{riu2}$, which is invariant to monotonic transformations of the gray scale and to rotation, and it is defined as:

(2) $$LBP_{P,R}^{riu2}=\left\{\begin{array}{cc} \sum_{p=0}^{P-1}s\left(g_{p}-g_{c}\right) & \mathrm{if}\;\;\;U(LBP_{P,R})\leq 2\\ P+1 & \mathrm{otherwise} \end{array}\right.$$

where

(3) $$\begin{array}{cc} U(LBP_{P,R}) & =|s\left(g_{P-1}-g_{c}\right)-s\left(g_{0}-g_{c}\right)|\\ & +\sum_{p=1}^{P-1}\left|s\left(g_{p}-g_{c}\right)-s\left(g_{p-1}-g_{c}\right)\right| \end{array}$$

There are only P+1 uniform patterns *U* (“pattern”), which are defined as the ones presenting a number of bit-wise transitions less than or equal to 2, in a neighbor of *P* pixels. On the other hand, all non-uniform patterns are labelled under the same category. Finally, a histogram of P+2 bins is built by computing $LBP_{P,R}^{riu2}$ for each pixel of the image, yielding the feature set of the image. In this work, we use $LBP_{P,R}^{riu2}$ but, for simplicity, we call it LBP henceforth.

#### 2.1.2. Adaptive Local Binary Patterns (ALBP)

ALBP [31] was motivated by the lack of information about the orientation in LBP. It takes into account the mean and the standard deviation along different orientations over all the pixels in order to improve the robustness against changes in the local spatial structure at the matching step. Guo et al. proposed a scheme to minimize the directional differences between the gray levels of the concerned pixels. This scheme allows softening the variations of the mean and standard deviation of the directional differences. The objective function is defined as follows:

(4) $$w_{p}=arg_{w}\min\left\{\sum_{i=1}^{N}\sum_{j=1}^{M}{\left|g_{c}\left(i,j\right)-w\cdot g_{p}\left(i,j\right)\right|}^{2}\right\}$$

where $w_{p}$ is the weight element used to minimize the directional difference, *w* is in the range from 0 to the maximum gray level value difference, and N and M are the number of rows and columns in the image respectively. Each weight $w_{p}$ is estimated along one orientation 2pπ/*P* for the whole image.

The ALBP output is defined as:

(5) $$ALBP_{P,R}=\sum_{p=0}^{P-1}s\left(g_{p}-w_{p}\cdot g_{c}\right)2^{p}\;,\;s\left(x\right)=\left\{\begin{array}{cc} 1 & \mathrm{if}x\geq 0\\ 0 & \mathrm{if}x<0 \end{array}\right.$$

In this paper, we compute ALBP using the uniform rotation invariant approach explained in Section 2.1.1, $ALBP_{P,R}^{riu2}$.

#### 2.1.3. Local Binary Patterns Variance (LBPV)

LBPV [32] combines LBP and a contrast distribution method. First, the uniform LBP is calculated in the whole image. Then, the variance of the image is used as an adaptive weight to adjust the contribution of the LBP code in the histogram calculation. The LBPV histogram is computed as:

(6) $$LBPV_{P,R}\left(k\right)=\sum_{i=1}^{N}\sum_{j=1}^{M}w\left(LBP_{P,R}\left(i,j\right),k\right),k\in \left[0,K\right]$$

where *k* represents a bin of the histogram, *K* the maximum value of LBP and *w* is defined as:

(7) $$w\left(LBP_{P,R}\left(i,j\right),k\right)=\left\{\begin{array}{cc} VAR_{P,R}\left(i,j\right), & LBP_{P,R}\left(i,j\right)=k\\ 0 & \mathrm{otherwise} \end{array}\right.$$

$VAR_{P,R}$ is the variance of the neighborhood.

(8) $$VAR_{P,R}=\frac{1}{P}\sum_{p=0}^{P-1}{\left(g_{p}-u\right)}^{2}$$

where *u* represents the mean over the different neighbors:

(9) $$u=1/P\sum_{p=0}^{P-1}g_{p}$$

In this work, we calculate the uniform rotation invariant LBPV, $LBPV_{P,R}^{riu2}$.

#### 2.1.4. Completed Local Binary Patterns (CLBP)

A local region is represented by its center pixel and a Local Difference Sign—Magnitude Transform (LDSMT). LDSMT decomposes the local structure of an image into two complementary components: the difference signs and the difference magnitudes. In order to code both components, Guo et al. [33] introduced two operators, CLBP-Sign (CLBP_S) and CLBP-Magnitude (CLBP_M). We concatenate both operators to form the final CLBP histogram. CLBP_S is identically defined as the original LBP in Equation (1), and CLBP_M is defined in Equation (10).

(10) $$CLBP\_M_{P,R}=\sum_{p=0}^{P-1}t\left(m_{p},c\right)2^{p}\;,\;t\left(x,c\right)=\left\{\begin{array}{cc} 1 & \mathrm{if}x\geq c\\ 0 & \mathrm{if}x<c \end{array}\right.$$

where *c* is a threshold that we set to the mean value of the differences between the central pixel and its neighbors, following [33].

In this paper, we use the uniform rotation invariant CLBP, $CLBP_{P,R}^{riu2}$. Note that Guo et al. also presented a third operator CLBP-Center (CLBP_C) that extracts the image local gray level but, for simplicity, we have not used it in this work.

## 3. Method

In this section, we describe in detail the booster that we propose, CLOSIB. Then, we present different CLOSIB variants which are very interesting in terms of accuracy (M-CLOSIB and HM-CLOSIB) and in terms of computational cost (H-CLOSIB). We include a summary of the notation used in Section 3 in Table 2.

### 3.1. Overview

**Figure 1:**
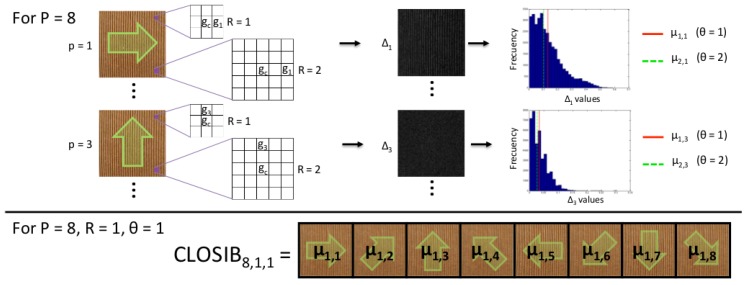
Overview of Complete Local Oriented Statistical Information Booster (CLOSIB) method. Example about the calculation of CLOSIB${}_{8,1,1}$ on an image.

In this subsection we present a brief description of CLOSIB with the support of Figure 1. Let us consider a given relative position of a pixel in the image with respect to the central pixel, for example the pixel that is placed to the right (*P* = 1) next to (*R* = 1) the central pixel. The absolute differences of the gray-scale values of pixels placed at a given position with respect to a central pixel $|g_{1}-g_{c}|$ are computed and stored at the position of the central pixel. This operation is done for every pixel of the image being considered as the central pixel of the image, which outputs the image $\Delta_{1}$. The values of $\Delta_{1}$ are represented in a histogram of absolute differences for a given relative position. Then, some statistical measure is computed on the histogram (mean, standard deviation). CLOSIB descriptor is made up of the statistical measures obtained when considering a set of relative positions around the central pixels.

LBP-based descriptors describe the texture of gray-scale images extracting their local spatial structure, whereas CLOSIB extracts statistical information of the gray-scale differences of an image. Thus, the nature of the information provided by LBP-based descriptors is completely different to the one provided by CLOSIB. This difference can be clearly noticed since LBP-based descriptors are local descriptors of the image, but CLOSIB is a global descriptor. Up to our knowledge, this is the first time the statistical information of the image is exploited on the basis of LBP approach.

### 3.2. Complete Local Oriented Statistical Information Booster (CLOSIB)

We propose a new enhancer that we name Complete Local Oriented Statistical Information Booster (CLOSIB). CLOSIB aims at improving the description performance of image feature descriptors.

CLOSIB is computed from the statistical information of the gray-scale differences of each pixel of the image. The gradient information of an image has been used in several texture descriptors in state-of-the-art. However, the statistical information of the gray-levels differences is infrequently taken into account for the description of an image. CLOSIB is conceptually simple and straightforward to implement.

Let us consider an image *I*, a particular pixel c∈I and a circularly symmetric set $N=\left\{p|p\in \left[1,\ldots ,P\right]\right\}$ where each *p* represents an equally spaced bearing around *c*. Let $g_{c}$ and $g_{p}$ be the gray values of pixel *c* located at $\left(x_{c},y_{c}\right)$ and its neighbor pixel $\left(x_{p},y_{p}\right)$ at bearing *p* on a circle of radius *R* respectively. Equation (11) states this relationship between $g_{c}$ and $g_{p}$ explicitly.

(11) $$\left(x_{p},y_{p}\right)=\left(x_{c}+R\cos\left(2\pi p/P\right),y_{c}-R\sin\left(2\pi p/P\right)\right)$$

The gray value of neighbors that are not located in the centers of pixels is estimated by interpolation of their connected pixels.

We define $\Delta_{p}$ as the absolute difference image at bearing *p*:

(12) $$\Delta_{p}=\left[|g_{c}-g_{p}|\right],\forall g_{c}\in I$$

**Figure 2:**
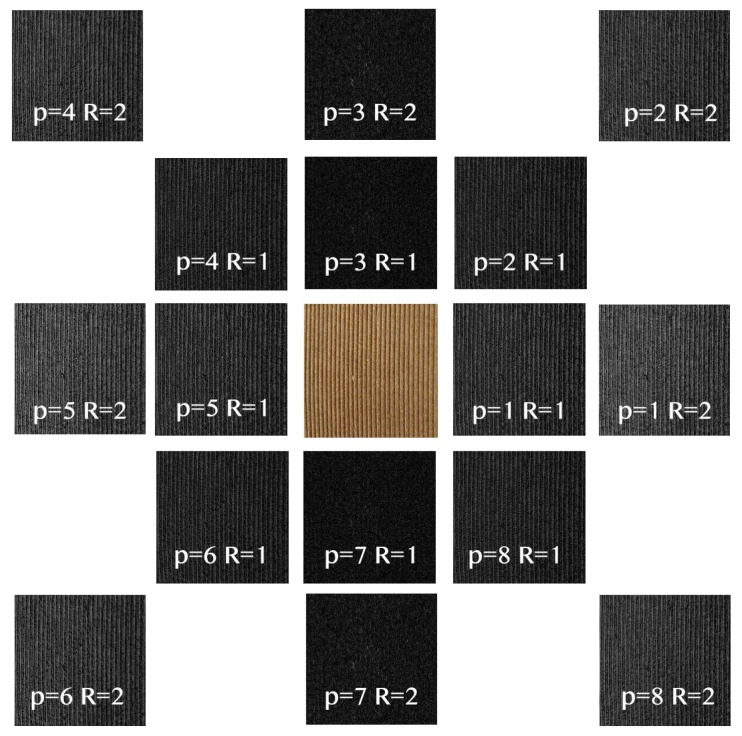
$\Delta_{p}$ images showing the absolute differences of the gray values for P=8 orientations in a neighborhood of radii R=1 and R=2. The original image *I* is shown in the center. The main change in the intensity of the original image occurs in the horizontal direction p=1 and p=5.

Figure 2 shows an example of the $\Delta_{p}$ images representing the absolute differences of the gray values for P=8 orientations in a neighborhood of radii R=1 and R=2.

Let $\mu_{i,p}$ represent the i-th moment of image $\Delta_{p}$:

(13) $$\mu_{i,p}=\frac{1}{N}\sum_{\forall g_{c}\in I}|g_{c}-g_{p}{|}^{i}$$

where *N* represents the number of pixels of image *I*.

We define the CLOSIB vector of image *I* for *P* bearings on a circle of radius *R* and θ-th moment:

(14) $$CLOSIB_{P,R,\theta }=\overset{P/\eta }{\underset{p=1}{∥}}{\left(\left(\theta -1\right)\mu_{2,p}-{\left(-1\right)}^{\theta }{\left(\mu_{1,p}\right)}^{\theta }\right)}^{1/\theta }$$

where ‖ represents the concatenation function, θ∈{1,2} is the order of the statistical moment considered, and η is a factor that controls the portion of the considered orientations in the quantized angular space. If not specified, we set η=1. Therefore, CLOSIB is a feature set of dimensionality P/η.

CLOSIB allows to adjust three parameters: the order of the statistical moment θ, the radius of the neighborhood *R* and the quantization of the angular space *P*.

The order of the statistical moment, θ, determines the statistical measure that is used to compute CLOSIB. For θ=1, CLOSIB is a feature set whose elements are the means of the $\Delta_{p}$ images representing the absolute differences of the gray values for each orientation and every pixel in the image. In the case of θ=2, the elements of CLOSIB are the standard deviations of the $\Delta_{p}$ images.

Parameter *R* determines the spatial resolution of the booster. Small radii are quite useful in images with a high level of heterogeneity. As the size of the neighborhood increases, noise is reduced but at the expense of a possible loss of valuable information, especially in images with high variability of the pixel values.

*P* controls the quantization of the angular space. A higher value of *P* means that a greater number of orientations are considered in the computation of CLOSIB. As the texture becomes more heterogeneous, the number of orientations should increase in order to capture all the variety of the image. However, using an excessive number of orientations on homogeneous textures may be counter-productive due to the loss of weight of the important ones.

### 3.3. CLOSIB Variants

In the literature, LBP is typically computed for (P,R) pairs of values equals (8,1), (16,2) or a concatenation of both. Likewise CLOSIB can be computed for (P,R,θ) triples of values equals (8,1,1), (8,1,2), (16,2,1), (16,2,2) or a concatenation of several of them. CLOSIB can also be computed for any other triple of values. We indicate the concatenation of several CLOSIBs with the symbol ‖. For example, the concatenation of CLOSIB${}_{8,1,1}$ and CLOSIB${}_{16,2,1}$ is represented as CLOSIB${}_{8,1,1∥16,2,1}$.

In this section, we propose and describe three specific ways of obtaining CLOSIB.

#### 3.3.1. Multi-Scale CLOSIB (M-CLOSIB)

Chang et al. in [45] proposed a multi-scale LBP (MSLBP) method for face detection that benefits from the multi-resolution information captured from the regional histogram. MSLBP has been extended and applied to other fields in the literature [46,47].

**Figure 3:**
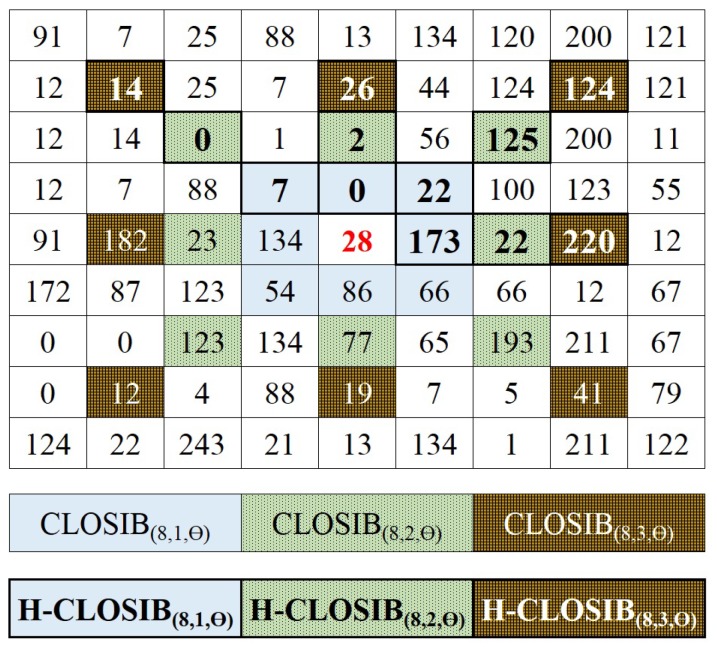
(Better viewed in color) Neighborhood around a center pixel, (28), considered for the computation of M-CLOSIB${}_{8,1,\theta ∥8,2,\theta ∥8,3,\theta }$ and HM-CLOSIB${}_{8,1,\theta ∥8,2,\theta ∥8,3,\theta }$. CLOSIB considers *P* = 8 orientations, while H-CLOSIB only *P* = 4 orientations. In the figure, neighbour pixels considered for H-CLOSIB are shown in bold.

Similarly, we introduce a multi-scale CLOSIB which we name M-CLOSIB. M-CLOSIB is a concatenation of the CLOSIBs obtained for a fixed number of orientations *P* and several radii of the neighborhood *R*. Figure 3 shows an schema of the computation of M-CLOSIB${}_{8,1,\theta ∥8,2,\theta ∥8,3,\theta }$, which results from the concatenation of CLOSIB${}_{8,1,\theta }$, CLOSIB${}_{8,2,\theta }$ and CLOSIB${}_{8,3,\theta }$.

#### 3.3.2. Half CLOSIB (H-CLOSIB)

**Figure 4:**
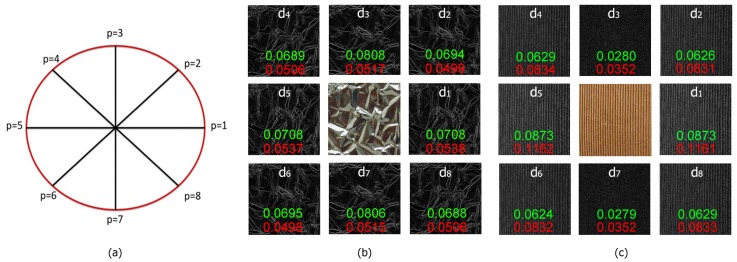
(**a**) Circumference that represents the neighborhood considered for the computation of CLOSIB with P=8. Four pairs of neighbors differ in π radians, such as the neighbors for values p=2 and p=6. (**b**,**c**) Schemas that represent the computation of CLOSIB${}_{8,1,\theta }$ for two different images. We show the original image in the centre and the eight images of the absolute differences of the gray values $d_{p}\left(x_{c},y_{c}\right)$ in the outer layer. The red and green numbers indicate the values of each element of CLOSIB${}_{8,1,1}$ and CLOSIB${}_{8,1,2}$ feature set, respectively, obtained for the corresponding *p* values of $d_{p}\left(x_{c},y_{c}\right)$. Note that the values of the elements of CLOSIB computed for neighbors that differ in π radians diverge in only a maximum of 0.0002 units whereas the ones that differ in a different angle diverge in at least 0.0006 units.

For even values of *P*, CLOSIB encompasses statistical information of the absolute differences of the gray values $d_{p}\left(x_{c},y_{c}\right)$ along directions that differ in π radians. Figure 4a shows this fact for P=8. The statistical information along directions that differ in π radians is usually very similar. Figure 4b,c illustrates two examples.

**Figure 5:**
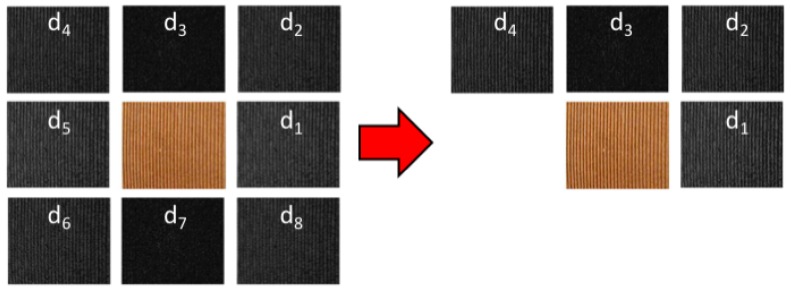
Schemas of the computation of CLOSIB${}_{8,1,\theta }$ (**left**) and H-CLOSIB${}_{8,1,\theta }$ (**right**) using the example of Figure 4c.

We define a Half CLOSIB (H-CLOSIB) following Equation (14) with η=2. The angular space is yet quantized in *P* equal parts but only the first P/η orientations are taken into account for the computation of H-CLOSIB. Figure 5 shows an example of the orientations considered when computing CLOSIB${}_{8,1,\theta }$ and H-CLOSIB${}_{8,1,\theta }$.

**Figure 6:**
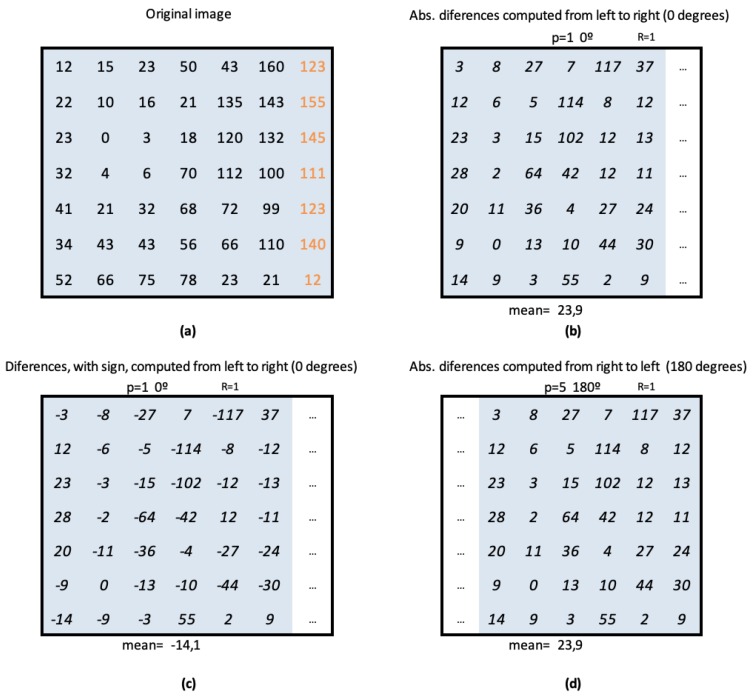
(**a**) Example of gray values of an Original Image. (**b**) Matrix obtained from the first difference of the gray values at 0 degrees. It corresponds with the first element of CLOSIB${}_{8,1,1}$. (**d**) Matrix obtained from the first difference of the gray values at 180 degrees. It corresponds with the fifth element of CLOSIB${}_{8,1,1}$. (**c**) Differences, with sign, at 0 degrees. CLOSIB uses the absolute value of the differences, therefore these values with sign are never computed.

H-CLOSIB presents two main characteristics. First, it may eliminate redundant statistical information. As the algorithm computes the magnitude of the first derivative, without sign, the absolute value of the differences between any pair of pixels is the same, without matter the direction of the gray-levels. In Figure 6, can be seen an example that allows to understand this fact better. In Figure 6a, the gray level values of the original image can be found. In Figure 6b,d are the values obtained applying p=1 and R=1 in the first case and p=5 and R=1 in the second. As can be seen, the value of the first order moment, denoted in this Figure as *mean* is the same in both cases, 23.9. Figure 6c presents the same calculation as in (b) but keeping the sign of the derivative. In this case, the mean has a different value of −14.1. The second characteristic is that the size of H-CLOSIB is half of the equivalent CLOSIB. The dimensionality might be decisive in some cases when the amount of memory or computational time are critical, such as in embedded systems with little RAM.

#### 3.3.3. Half Multi-Scale CLOSIB (HM-CLOSIB)

We propose a Half Multi-scale CLOSIB (HM-CLOSIB) which is obtained as a M-CLOSIB when η=2. This variant combines the advantages and disadvantages of both M-CLOSIB and H-CLOSIB. Figure 3 shows a schema of the computation of HM-CLOSIB${}_{8,1,\theta ∥8,2,\theta ∥8,3,\theta }$, which results of the combination of H-CLOSIB${}_{8,1,\theta }$, H-CLOSIB${}_{8,2,\theta }$ and H-CLOSIB${}_{8,3,\theta }$.

## 4. Experiments and Results

### 4.1. Datasets

#### 4.1.1. KTH TIPS2-a

KTH TIPS2-a dataset (http://www.nada.kth.se/cvap/databases/kth-tips/download.html) aims at evaluating algorithms for classifying materials [15]. It includes a total of 4608 images grouped into 11 classes. The dataset contains four physical samples of each of the 11 materials. The dataset presents a high intra-class variation regarding texture and colour. All samples were taken at nine scales and three poses under four different illumination conditions, which makes the dataset very challenging.

#### 4.1.2. UIUC

The University of Illinois Urbana-Champaign (UIUC) texture dataset (http://www-cvr.ai.uiuc.edu/ponce_grp/data/index.html) [41] contains 25 different texture classes within 40 images per class, giving a total 1000 un-calibrated, unregistered gray-scale images of resolution 640 × 480 pixels. The database contains materials, fabrics and other textures such as water. Within each class, significant viewpoints variations, scale changes and non-rigid deformations are strongly present [2]. This dataset contains a few numbers of images per class but a high intra-class variability, being a challenging dataset regarding scale and other viewpoint variations.

#### 4.1.3. USPTex

USPTex dataset (http://fractal.ifsc.usp.br/dataset/USPtex.php) [42] contains 191 different classes of 24-bit color png images of general scenes like roads, vegetation, walls, clouds and materials such as seeds, rice or tissues. The most challenging feature of this dataset is the low number of images per class (12), their low resolution (128×128 pixels) and the high number of classes included [3].

#### 4.1.4. JAFFE

JAFFE dataset (http://www.kasrl.org/jaffe.html) [22] comprises 213 images of 7 facial expressions (6 basic facial expressions and 1 neutral) posed by 10 Japanese female models. Each subject appears in 20 to 23 images. The images were taken from a frontal pose, and tungsten lights were used to create even illumination on the face. All images are 256×256 pixels in size. In this paper, we use JAFFE dataset for face recognition instead of expression recognition. Therefore, we are dealing with a multiclass classification that comprehends 10 classes.

### 4.2. Experimental Setup

For KTH-TIPS 2a dataset, we used the experimental protocol developed by Caputo et al. [15,16], which is 4-fold cross-validation along the samples of each material. For each fold, we used all images of one sample of each material for testing and the rest for training. This experimental setup is more challenging than a random division of the images into training and test sets due to the high inter-sample variation. We report the results as the average hit rate over the four runs. We define the hit rate as the number of correctly classified images divided by the total number of images in the test set. We used a Support Vector Machine (SVM) to classify the images with the Least Squares training algorithm and a polynomial kernel of order 2. We used the one-vs-one paradigm [48] in which n(n−1)/2 binary classifiers are trained for a *n*-way multi-class problem; each receives the samples of a pair of classes. For testing, all binary classifiers are applied to an unseen sample, and the class that gets the highest number of predictions for all binary classifiers gets predicted.

Concerning UIUC and USPTex datasets, we carried out random sub-sampling cross-validation with 10 repetitions to avoid overfitting. In each iteration, the model is fit to a training set of 75% of the images, and predictive accuracy is assessed using the rest of the images. The results were averaged over the splits. We used an SVM trained using Least Square algorithm and a linear kernel.

Regarding JAFFE dataset, we used the same evaluation setup proposed by Sharma et al. [49]. Specifically, one random image of each facial expression and person forms the test set, and the rest define the training set. We repeat the classification 10 times to avoid biased results due to the random process. We used the multi-block approach introduced by Zang et al. [50] for describing a face using LBP-based descriptors and CLOSIB. We split the image into 8×8 blocks and compute a descriptor for each block. We define the descriptor of the image as the concatenation of the descriptors of the blocks. We performed two sets of experiments with JAFFE dataset. On the one hand, we used the images provided in the dataset. On the other hand, we automatically cropped the face of the images using Viola-Jones method [51] and used the cropped images to carry out the experiments. Table 3 and Table 4 show the CLOSIBs used in the experiments for CLOSIB and H-CLOSIB, and M-CLOSIB and HM-CLOSIB, respectively.

In the following subsections, we present and discuss the results obtained using this experimentation. We aim to check if CLOSIB enhances the performance of LBP-based descriptors on several public texture datasets for different fields. For KTH Tips2-a, a more thorough review of the performance of CLOSIB is introduced in order to better understand its behavior.

### 4.3. Results for KTH Tips2-a

#### 4.3.1. CLOSIB versus LBP-Based Descriptors

**Figure 7:**
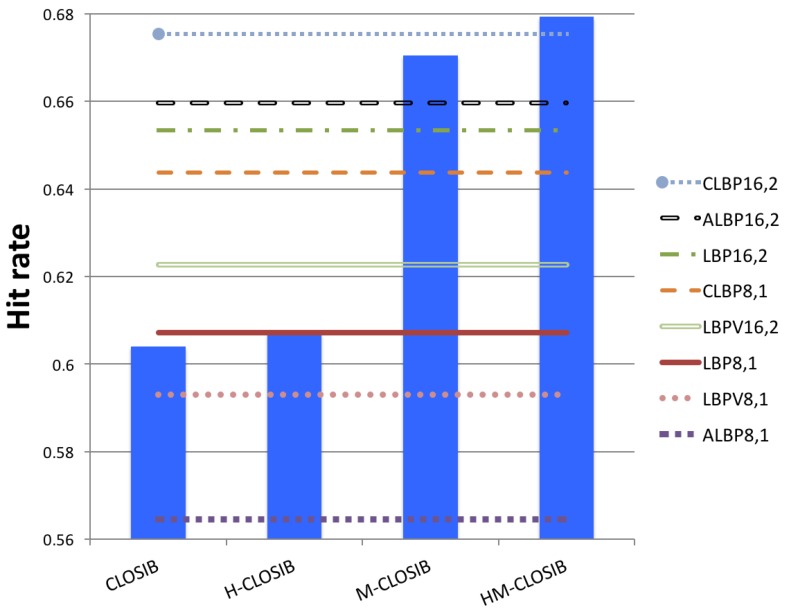
Hit rates when we describe KTH Tips2-a images with different CLOSIBs and LBP-based descriptors. For each CLOSIB variant –CLOSIB (standard), M-CLOSIB, H-CLOSIB and HM-CLOSIB–, we only represent the best result obtained among the results with different combinations of parameters.

We developed CLOSIB as an enhancer of texture descriptors. However, in this section, we show the performance of CLOSIB as a descriptor itself. Figure 7 presents the results that we obtained when describing the images with CLOSIB and with descriptors based on LBP. For all CLOSIB variants, we achieved the best performance using a concatenation of the CLOSIBs for the first and second statistical moments. For all LBP-based descriptors, we obtained the best results for R=2 pixels and P=16 neighbors. It is remarkable that we achieved the highest performance using HM-CLOSIB${}_{16,2,1∥16,2,2∥16,3,1∥16,3,2∥16,4,1∥16,4,2}$ which yielded a hit rate of 67.93%. Therefore, the proposed enhancer by itself outperforms some of the state-of-the-art LBP-based descriptors.

#### 4.3.2. CLOSIB and LBP-Based Descriptors

**Figure 8:**
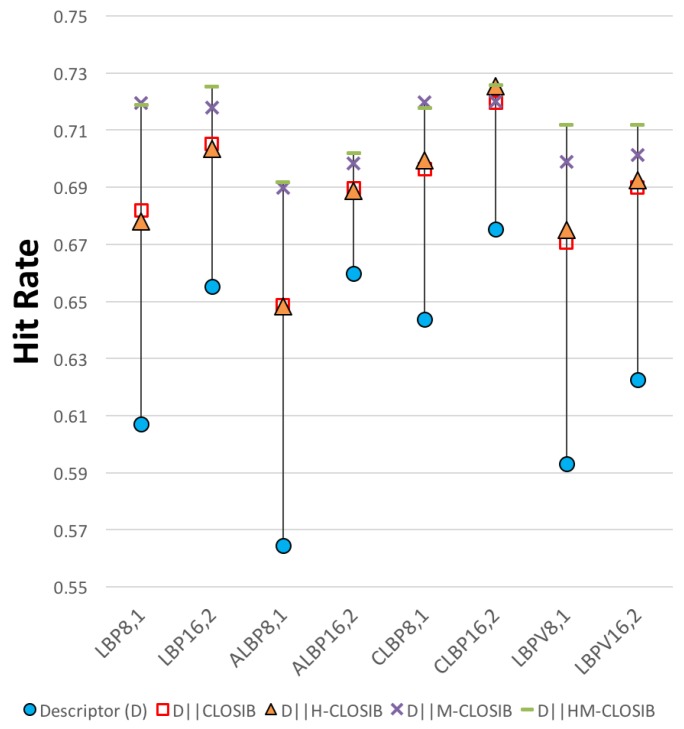
Hit rates obtained with a given LBP-based descriptor (LBP, ALBP, LBPV and CLBP) and the concatenations of the descriptor with CLOSIB variants.

The following experiment consists of combining LBP-based descriptors with CLOSIB. The combination is done by means of a concatenation. Figure 8 and Table 5 graphically and numerically show the results.

In all experiments, we achieved the best results using CLOSIB as an enhancer of LBP-based descriptors in opposition to only using LBP-based descriptors. We obtained the highest hit rates equal to 72.54% with CLBP${}_{16,2}∥$HM-CLOSIB${}_{16,2,1∥16,2,2∥16,3,1∥16,3,2∥16,4,1∥16,4,2}$ and CLBP${}_{16,2}∥$H-CLOSIB${}_{16,2,1∥16,2,2}$ closely followed by LBP${}_{16,2}∥$HM-CLOSIB${}_{\begin{array}{c} 8,1,1∥8,1,2∥8,2,1∥8,2,2∥8,3,1\\ ∥8,3,2∥8,4,1∥8,4,2∥8,5,1∥8,5,2 \end{array}}$ with a hit rate of 72.50%. For 6 out of the 8 LBP-based descriptors, we achieved the best results with the concatenation of HM-CLOSIB.

#### 4.3.3. No Multi-Scale versus Multi-Scale LBP-Based Descriptors

**Figure 9:**
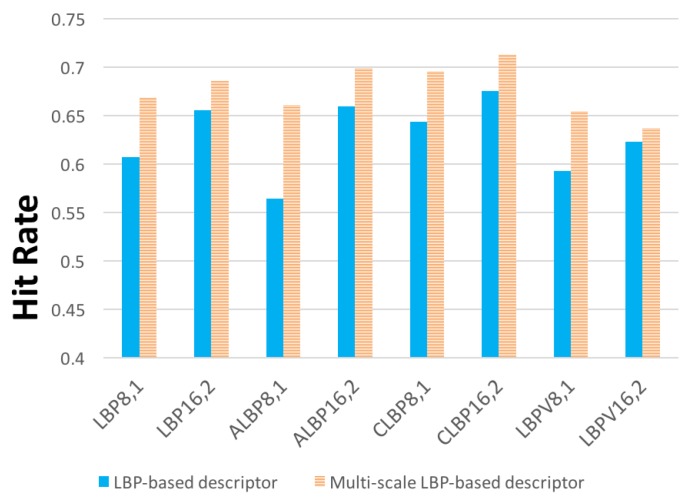
Hit rates for LBP-based descriptors LBP, ALBP, CLBP and LBPV and their multi-scale versions.

The good performance of multi-scale CLOSIB leads us to reproduce the experiments for multi-scale LBP-based descriptors. Figure 9 shows the comparison between the results obtained with LBP-based descriptors and their multi-scale versions.

We defined a multi-scale LBP as a concatenation of the LBP descriptors obtained with different neighborhood radii (*R*) and the same number of neighbors (*P*). We used R={1,2,3} for P=8 and R={2,3,4} for P=16. The hit rate fairly improves with multi-scale LBP-based descriptors in all cases. Therefore, multi-scale descriptors are very interesting for texture retrieval.

Best result was obtained with CLOSIB${}_{16,2∥16,3∥16,4}$ with a 71.28%. This result means a 5.55% of improvement compared with the standard CLOSIB${}_{16,2}$. However, our proposed descriptor HM-CLOSIB combined with CLBP${}_{16,2}$ gets the best performance so far.

#### 4.3.4. HM-CLOSIB + Multi-Scale LBP-Based Descriptors

Finally, we evaluated the combination of multi-scale LBP-based descriptors with HM-CLOSIB. We selected HM-CLOSIB due to the high performance achieved in terms of accuracy and computational time in previous experiments.

**Figure 10:**
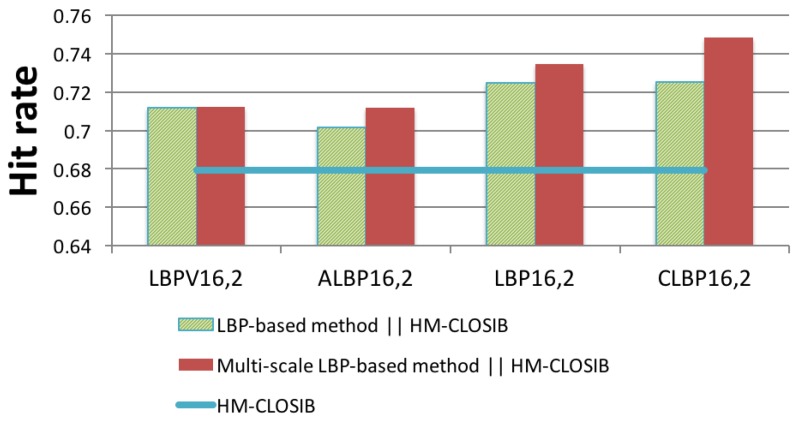
Hit rates obtained with the concatenation of multi-scale LBP-based descriptors and HM-CLOSIB. The horizontal line represents the hit rate of HM-CLOSIB descriptor.

Figure 10 shows the hit rate of the concatenation of multi-scale LBP-based descriptors with HM-CLOSIB. Furthermore, we also present the hit rate of the (non multi-scale) LBP-based descriptors combined with HM-CLOSIB to represent the improvement in accuracy.

CLBP${}_{16,2}∥$HM-CLOSIB${}_{16,2,1∥16,2,2∥16,3,1∥16,3,2∥16,4,1∥16,4,2}$ outperformed the rest of the methods with a hit rate of 74.83%, which represents an improvement of at least 3.16% in hit rate with respect to the rest of descriptors.

#### 4.3.5. Comparative with the State-of-the-Art

Several authors tested their algorithms using KTH TIPS2-a dataset. In Table 6, we can see the results achieved by 23 state-of-the-art methods, including three that are based on deep learning approaches. To the best of our knowledge, the best result has been yielded by by LFV+FC-CNN [52], an approach where deep features are extracted. The second and third positions are obtained by another deep features approach NmzNet [53], followed by a handcrafted one, IFV [54].

The classification performance of the proposed descriptor CLBP${}_{16,2}∥$HM-CLOSIB${}_{16,2,1∥16,2,2∥16,3,1∥16,3,2∥16,4,1∥16,4,2}$ is the fourth over the 20 methods based on handcrafted approaches. Furthermore, using just the straightforward HM-CLOSIB as a descriptor, we yielded a higher hit rate than most of these studies.

Note that a direct comparison among the results reported by these methods cannot be made due to the different approaches that were taken for preprocessing the images—here no preprocessing has been done—and for carrying out the experiments. It can be found that our booster achieves comparable results to those of the state-of-the-art and that it can be successfully used in combination with LBP-based methods to enhance their performance. As we mentioned in Section 1, we published results using CLOSIB booster together with HOG features [43], demonstrating that CLOSIB could be successfully combined with several handcrafted features, not only LBP-based methods.

### 4.4. Results for UIUC and USPTex

**Figure 11:**
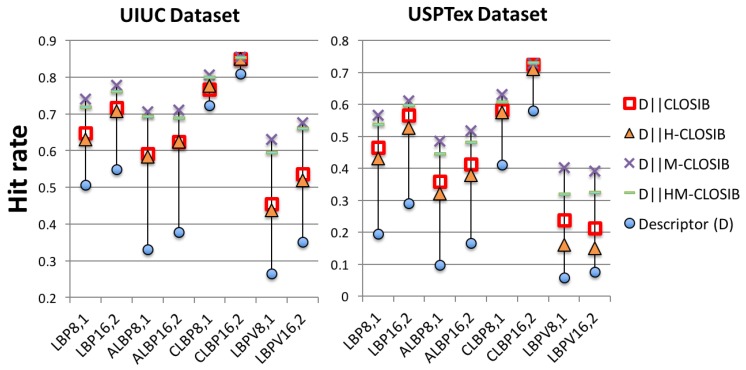
Results using the concatenation of LBP-based descriptors with CLOSIB variants (CLOSIB, H-CLOSIB, M-CLOSIB and HM-CLOSIB) on UIUC (**left**) and USPTex (**right**) dataset.

Figure 11 shows the hit rates that we obtained on UIUC and USPTex datasets, respectively. In both cases, every combination of LBP-based descriptors with any CLOSIB variant yielded higher hit rates than the LBP-based descriptors alone. M-CLOSIB outperformed the rest of CLOSIB variants. For UIUC, we obtained the highest hit rate (85.51%), whereas for USPTex, we achieved the highest hit rate (72.91%), in both cases using CLBP${}_{16,2}∥$M-CLOSIB${}_{16,2,1∥16,2,2∥16,3,1∥16,3,2∥16,4,1∥16,4,2}$.

### 4.5. Results for JAFFE

We carried out two sets of experiments with JAFFE dataset: with the original images and with automatically cropped images.

**Figure 12:**
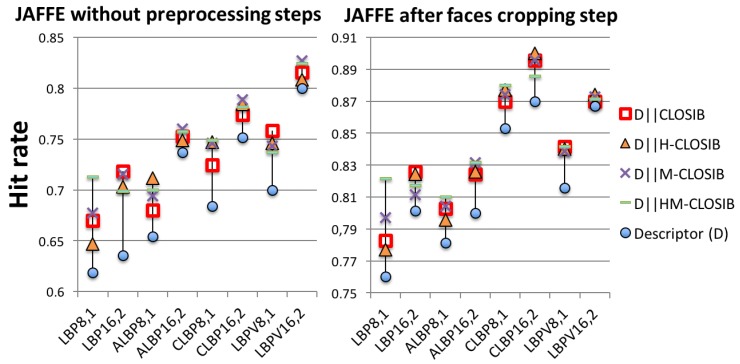
Results using the concatenation of LBP-based descriptors with CLOSIB variants (CLOSIB, H-CLOSIB, M-CLOSIB and HM-CLOSIB) on the original images (**left**) and the cropped ones (**right**) of JAFFE dataset.

Figure 12 shows the hit rates that we obtained in the first experiment, using the original images. In all cases, the LBP-based descriptors achieved worse results than the combination of the LBP-based descriptors with any CLOSIB variant. The combination with M-CLOSIB yielded the highest hit rates in most of the cases, except for LBP${}_{16,2}$ and LBPV${}_{8,1}$ in which the combination with CLOSIB outperformed the others. We achieved the best results using ${\mathrm{LBPV}}_{16,2}∥{M-\mathrm{CLOSIB}}_{\begin{array}{c} 8,1,1∥8,1,2∥8,2,1∥8,2,2∥8,3,1\\ ∥8,3,2∥8,4,1∥8,4,2∥8,5,1∥8,5,2 \end{array}}$ with a hit rate of 82.71%.

Regarding the second experiment, Figure 12 shows the hit rates achieved using the cropped images. Again, tests using LBP-based descriptors yielded worse results than when combined with any CLOSIB variant. In this case, we obtained the highest hit rate, 90.00%, using ${\mathrm{CLBP}}_{16,2}∥{H-\mathrm{CLOSIB}}_{16,2,1||16,2,2}$. It is important to notice that carrying out the preprocessing step, the performance improves up to 8.81%.

### 4.6. Computational Cost of CLOSIB and LBP Variants

Finally, we present in Table 7 and Table 8 the average computational time per image employed for the extraction of CLOSIB and LBP variants descriptors, respectively, on the four datasets studied. The fastest descriptors per dataset are shown in bold.

In Table 7 we can observe how LBP${}_{8,1}$ is the fastest choice for UIUC and USPTex datasets, with 0.09183 and 0.00351 seconds per image respectively, while CLBP${}_{8,1}$ is for KTH TIPS2-a and JAFFE with 0.00754 and 0.01013 seconds, respectively. In Table 8, it can be noticed that CLOSIB variants require similar or even less computational time than the LBP variants for equal values of neighbors and order. Regarding CLOSIB variants, the shortest times are achieved by H-CLOSIB${}_{1,8,1}$ proposal, obtaining an average of 0.0863, 0.00377, 0.00838 and 0.01442 seconds per image on UIUC, USPTex, KTH TIPS2-a and JAFFE datasets, respectively.

## 5. Conclusions

We proposed a new texture descriptor booster, called CLOSIB, which is based on the statistical information provided by the gray-level differences of the image. Furthermore, we presented three variants of CLOSIB: H-CLOSIB, useful for embedded systems or machines with a low RAM; M-CLOSIB, a multi-scale descriptor which extracts information for consecutive neighborhoods; and HM-CLOSIB, which is a multi-scale H-CLOSIB. The experiments demonstrated that H-CLOSIB is a little bit more efficient than the general version in terms of precision and computational cost. We also saw that a description of the image at several scales, using the M-CLOSIB, always produces comparable or better results than the general version of CLOSIB. Those differences are very significative in some of the used datasets. We evaluated CLOSIB in three applications: material recognition using KTH TIPS2-a dataset, general texture recognition using UIUC and USPTex datasets and face recognition using JAFFE dataset.

Regarding material recognition, HM-CLOSIB outperformed some of the state-of-the-art LBP-based descriptors. To check the performance of CLOSIB as an enhancer of other texture descriptors, we used a concatenation of LBP-based descriptors with CLOSIB variants. All tested combinations of LBP-based descriptors with CLOSIB yielded better results than the individual descriptors. Moreover, we proved that the classification results for material recognition improves when using multi-scale LBP-based descriptors. We obtained the best result using a concatenation of a multi-scale CLBP and HM-CLOSIB yielding a hit rate of 74.83%. Finally, this method outperformed some relevant state-of-the-art methods tested on KTH TIPS2-a images. Concerning general texture recognition (UIUC and USPTex), every concatenation of LBP-based descriptors with CLOSIB variants yielded higher hit rates than the individual LBP-based descriptors. In relation to face recognition, the combination of LBP-based descriptors with CLOSIB variants outperformed the individual descriptors as well. We obtained the highest hit rate of 90% using a combination of CLBP${}_{16,2}$ and H-CLOSIB when automatically cropping the images of the dataset by means of the Viola-Jones method.

All in all, in this paper we introduced a new, efficient and powerful texture descriptor enhancer that adds statistical information about the gray-level differences of the pixels of the image employing a straightforward implementation. Based on the results obtained, we consider that CLOSIB can be regarded as a descriptor enhancer of broad purpose that, when fused with other descriptors, provides new and relevant information that improves the classification results.

In the future, we will evaluate the performance obtained when combining CLOSIB with other different texture descriptors to determine with which ones it works better and its limitations, if any. We also will propose a HM-CLOSIB for color images and we will evaluate how a rotational invariant codification performs. Among the methods used for pornography detection, skin detection approach uses texture descriptors [62]. In the context of the 4NSEEK European Project, we will explore how the combination of CLOSIB booster and texture descriptors affects the accuracy of a system used for porn detection, and more specifically, for the fight against Child Sexual Abuse (CSA).

## Figures and Tables

**Table 1:** Local Binary Patterns (LBP) variants notation.

Parameter	Meaning
$g_{c}$	Gray value of the central pixel
$g_{p}$	Gray value of neighbor *p*
*P*	Number of neighbors
*R*	Radius of the neighborhood
$w_{p}$	Weight element used to minimize the directional difference
*w*	Weight, it is a constant between 0 to the maximum gray level value difference
*N*	Number of rows in the image
*M*	Number of columns in the image
*k*	A bin of a histogram
*K*	Maximum value of LBP
*u*	Mean over the neighbors
*c*	Threshold, mean value of the differences between the central pixel and neighbors

**Table 2:** CLOSIB variants notation.

Parameter	Meaning
*I*	Image
*c*	Central pixel
*p*	Neighbor pixel
$g_{c}$	Gray value of pixel *c*
$g_{p}$	Gray value of neighbor pixel *p*
*R*	Radius of the neighborhood
$\Delta_{p}$	Absolute difference image at bearing *p*
$\mu_{i,p}$	i−th moment of image $\Delta_{p}$
‖	Concatenation function
θ	Order of the statistical moment considered
η	Variable that allows choosing between CLOSIB and H-CLOSIB

**Table 3:** Each row describes the parameters used to compute different CLOSIBs and H-CLOSIBs in the experiments. In column “order”, values 1 and 2 indicate that we obtained CLOSIB as a concatenation of the CLOSIBs for each statistical moment, $CLOSIB_{P,R,1∥P,R,2}$.

Radius (*R*)	Neighbors (Orientations) (*P*)	Order (θ)
1	8	1
1	8	2
2	16	1
2	16	2
1	8	1,2
2	16	1,2

**Table 4:** Each row describes the parameters used to compute different M-CLOSIBs and HM-CLOSIBs in the experiments. Several values for a parameter indicate that we obtained CLOSIB as a concatenation of the CLOSIBs for each single value.

Radius (*R*)	Neighbors (Orientations) (*P*)	Order (θ)
1,2,3	8	1
1,2,3,4,5	8	1
1,2,3	8	2
1,2,3,4,5	8	2
2,3,4	16	1
2,3,4,5,6	16	1
2,3,4	16	2
2,3,4,5,6	16	2
1,2,3	8	1,2
1,2,3,4,5	8	1,2
2,3,4	16	1,2
2,3,4,5,6	16	1,2

**Table 5:** Hit rates (in %) obtained with a given LBP-based descriptor (LBP, ALBP, LBPV and CLBP) and the concatenations of the descriptor with CLOSIB variants. The best results for each LBP-based descriptor are highlighted in bold. The best overall results are underlined. D stands for Descriptor and C for CLOSIB.

Descriptor (D)	D	D||C	D||H-C	D||M-C	D||HM-C
**LBP** ${}_{8,1}$	60.71	68.20	67.80	**71.95**	71.86
**LBP** ${}_{16,2}$	65.53	70.52	70.33	71.78	**72.50**
**ALBP** ${}_{8,1}$	56.46	64.86	64.84	68.97	**69.15**
**ALBP** ${}_{16,2}$	65.97	68.96	68.88	69.84	**70.16**
**LBPV** ${}_{8,1}$	59.30	67.05	67.51	69.89	**71.15**
**LBPV** ${}_{16,2}$	62.27	69.00	69.24	70.14	**71.17**
**CLBP** ${}_{8,1}$	64.37	69.63	69.95	**71.97**	71.76
**CLBP** ${}_{16,2}$	67.53	71.95	**72.54**	72.01	**72.54**

**Table 6:** Hit rates obtained by the proposed booster, the combination of the booster with CLBP and the reported classification scores for 18 state-of-the-art methods on the KTH-TIPS2-a. Scores are as originally reported. Our proposal is highlighted in bold, together with the best proposal of the Deep Features.

Descriptor—Handcrafted	Hit Rate (%)	Reference
**WLD**	56.4	[16]
**MWLD**	64.7	[16]
**SIFT**	52.7	[16]
**LTP**	60.7	[17]
**LQP**	64.2	[17]
**WLBP**	64.4	[55]
**LHS**	73.0	[49]
**CMLBP**	73.1	[56]
**CMR**	69.4	[57]
**PC**	71.5	[57]
**DRLTP**	62.6	[58]
**DRLBP**	59.0	[58]
**HoPS**	75.0	[59]
**IFV**	82.2	[54]
**AMBP**	70.3	[18]
**MS4C**	70.5	[60]
**CRDP** ${}_{3D}-2$ **(NNC)**	73.8	[61]
**CRDP** ${}_{3D}-2$ **(SVM)**	78.0	[61]
**HM-CLOSIB**	67.9	Ours
**CLBP** ${}_{16,2}∥$ **HM-CLOSIB**	**74.8**	Ours
**Descriptor—Deep Features**	**Hit Rate (%)**	**Reference**
**DeCAF**	78.4	[54]
**LFV + FC-CNN**	**82.6**	[52]
**NmzNet**	82.4	[53]

**Table 7:** Computational times, in seconds, for the extraction of LBP variants on the four datasets evaluated. LBP variants with underscored parameters *neighborhood*, *radius*.

Dataset	LBP${}_{8,1}$	LBP${}_{16,2}$	ALBP${}_{8,1}$	ALBP${}_{16,2}$	LBPV${}_{8,1}$	LBPV${}_{16,2}$	CLBP${}_{8,1}$	CLBP${}_{16,2}$
**UIUC**	**0.09183**	0.17636	0.20309	0,44195	0.15119	0.34011	0.1157	0.23574
**USPTex**	**0.00351**	0.00488	0.00605	0.01051	0.00428	0.00918	0.00361	0.00594
**KTH-TIPS2-a**	0.00914	0.01084	0.01297	0.02555	0.0114	0.02283	**0.00754**	0.01326
**JAFFE**	0.01116	0.01695	0.02179	0.04263	0.01628	0.03312	**0.01013**	0.0183

**Table 8:** Computational times, in seconds, for the extraction of CLOSIB variants on the four datasets evaluated. CLOSIB (**C**) and H-CLOSIB (**H-C**) with underscored parameters (*radius*, *neighbors*, *order*). M-CLOSIB (**M-C**) and HM-CLOSIB (**HM-C**) with underscored parameters (*minRadius*, *maxRadius*, *neighbors*, *order*).

Dataset	C${}_{1,8,1}$	C${}_{2,16,2}$	H-C${}_{1,8,1}$	H-C${}_{2,16,2}$	M-C${}_{1,3,8,1}$	M-C${}_{2,4,16,2}$	HM-C${}_{1,3,8,1}$	HM-C${}_{2,4,16,2}$
**UIUC**	0.08655	0.18975	**0.08630**	0.18975	0.25392	0.59675	0.25142	0.56396
**USPTex**	0.00393	0.00594	**0.00377**	0.00564	0.00938	0.01584	0.00912	0.01471
**KTH TIPS2-a**	0.00921	0.01782	**0.00838**	0.01682	0.02284	0.05158	0.02266	0.04846
**JAFFE**	0.02640	0.02790	**0.01442**	0.02594	0.03833	0.07177	0.03582	0.06767

## Abbreviations

The following abbreviations are used in this manuscript:

LBP	Local Binary Pattern
ALBP	Adaptive Local Binary Pattern
ALBPV	Adaptive Local Binary Pattern Variance
CLOSIB	Complete Local Oriented Statistical Information Booster
H-CLOSIB	Half Complete Local Oriented Statistical Information Booster
M-CLOSIB	Multi-scale Complete Local Oriented Statistical Information Booster
ASASEC	Advisory System Against Sexual Exploitation of Children
CDC	Compact Digital Cameras
CNN	Convolutional Neural Networks
CSA	Child Sexual Abuse
